# Aviophobia and its relationship to stress and epileptic-like symptoms: a narrative review

**DOI:** 10.3389/fpsyt.2026.1911877

**Published:** 2026-10-05

**Authors:** Matthew Laker, Petr Bob, Norbert Riethof

**Affiliations:** 1 Center for Neuropsychiatric Research of Traumatic Stress, Department of Psychiatry and UHSL, First Faculty of Medicine, Charles University, Prague, Czechia; 2 School of Art and Sciences, LaSalle University, Philadelphia, PA, United States; 3 Department of Psychology, UHSL, First Faculty of Medicine, Charles University, Prague, Czechia

**Keywords:** anxiety, aviophobia, epileptic-like symptoms, epileptiform activity, sensitization, stress

## Abstract

Travel by air and global mobility have become essential for an increasing number of people and, in many cases, may cause anxiety symptoms of aviophobia. Although variations exist in estimates of aviophobia prevalence, undeniably large numbers of people suffer consequences that influence their work and personal lives. Despite the existence of effective treatments, a high prevalence, above and below the clinical threshold, plus diagnostic difficulties may complicate therapeutic options for treating aviophobia. Based on current evidence, this article provides an overview of recent findings and future research and treatment perspectives, including novel findings indicating that certain stressful aviophobia experiences may be linked to temporal and limbic epileptiform activity, which may produce epileptic-like symptoms.

## Introduction

After World War I, a substantial percentage of combat pilots demonstrated strong aversive feelings towards flights, indicating severe psychological symptoms that also manifest physiologically, e.g., insomnia and gastric disturbances (1–4). Their treating physicians, unable to identify a physiological basis for their symptoms, instead designated them as being psychological in origin and introduced the diagnosis of “aero-neurosis” to characterize the disorder (1, 2, 4, 5).

As a clear variation in susceptibility among pilots and flight crew was evident, the propensity towards suffering from the disorder was attributed to “temperament or a family history of ‘nervous instability”. However, in subsequent peaceful times, interest in the disorder naturally subsided until World War II when the obviously renewed use of combat air power resulted in new cases of the disorder later called “aviophobia” or “fear of flying” (2–5). With subsequent developments from the 1970s to the present, these cognitive, affective, and behavioral symptoms of aviophobia have been primarily studied from the perspective of fear conditioning and cognitive behavioral therapy (2, 4–6).

## Symptoms of aviophobia, diagnostic criteria and epidemiology

According to the Diagnostic and Statistical Manual 5 (7), the fear of flying, “aviophobia”, falls within the specific phobia category. A typical aviophobic experience is a fear that is intense, distinctive, enduring, and clearly non-rational in proximity to a given stimulus: flight in an aircraft. The fear may be oriented towards the possibility of an aircraft crash causing potential harm (7). Cognitive responses of these patients towards flight may also include general fear of dying, fears of embarrassment, and fears of behaving uncontrollably (2, 4, 5, 8). Physiological responses, conversely, may include increases in heart rate and blood pressure, hyperventilating, ‘gastric distress,’ and frequent panic attacks (2, 4, 5). Anxiety is experienced and immediately felt when in contact with the phobic object, increases and decreases accordingly in response to distance from the phobic object, and may increase in the event of the potential denial of ‘escape routes’ from the phobic object. The sufferer would accordingly experience relatively low levels of anxiety during boarding, followed by correspondingly more severe anxiety during the middle point of a flight (7). A few specific distinctions would be worth noting. While the sufferers are often cognizant that their fear is non-rational, they will tend to view the risk of harm as much greater than the objective actuality, leaving the assessment of ‘disproportionate’ fear to a clinician’s judgment (7).

Additionally, within the context of aviophobia, a diagnosis would not be appropriate in the event of an existing situation which might reasonably justify the fear, such as being subjected to hostile bombardment or performing high-speed maneuvers near physical objects (7).

Van Gerwen et al. (9), noting the disorder’s diagnostic difficulties, suggest that the fear of flying is comprised of three components: cognitions; behavior and physiology. Highly useful, given this complexity, are two psychometric instruments, the Flight Anxiety Situations (FAS) questionnaire and the Flight Anxiety Modality (FAM) questionnaire, which are helpful in identifying and screening for these specific symptoms. Combined usage of these two instruments assists in identifying subtle differences in fear experienced by sufferers.

Currently, the Diagnostic and Statistical Manual 5 criteria require that experienced fear in response to the phobic stimulus must be irrational (7). Although it is obvious that both early experimental flight and air combat had the clear potential to result in death or severe injury (2–4, 10), contemporary air travel is widely known to be one of the safest activities which may be engaged in, statistically less risky than taking a train, driving, or even ‘staying at home’. Fewer than 2.5 incidents occur in every 1 million flight hours, and of these, fewer than 1 out of 10 results in a fatality (2, 8, 11). However, despite this near-impeccable record of safety, the disorder remains highly prevalent as previously discussed in the Diagnostic and Statistical Manual 5 (7).

For example, in 2008, using the then-current Diagnostic and Statistical Manual 3 criteria for specific/simple phobia, a random selection survey of 7076 adults in the Netherlands identified a lifetime prevalence of 2.5% (2, 4, 12). The previous year, a survey of 43093 adults in the United States using a stated criterion of “Excessive fear resulting in restricted activity assessed by interview with experienced lay interviewer” identified again a lifetime prevalence of 2.9% with “Specific phobias more prevalent among females, lower income groups and white participants than Asian or Hispanic” (2, 13). Using less stringent criteria, a survey of 2117 Americans asked, “Are you afraid of flying?” and found 17% answering “yes”. Additionally, even flight attendants and pilots who have chosen to make flying a part of their daily work lives report disproportionate fear relative to the well-understood risk (2, 4, 14). A survey distributed to 1147 aircrew in a European commercial airline indicated that slightly more than 9% experienced fear more than once a month and that almost 3% reported experiencing fear and anxiety on a weekly or even daily basis. However, it is noteworthy that most positive responses originate from the cabin crew. 80% of flight deck crew report ‘never’ being afraid. Higher prevalence was reported among female crew (2, 4, 15, 16). Interestingly, in turn, recent research has found a significant relationship between the perceived competence and service quality provided by the cabin crew and passenger in-flight anxiety (4).

The manifestations of aviophobia include a variety of symptoms, most observably when sufferers avoid flying, fly only when necessary, or exhibit a high degree of anxiety while in flight (2, 4, 8). Several safety compulsive behaviors may also be demonstrated, including strong preferences in seating (close to the exits or in window seats to avoid contact with other passengers) and making nervous inquiries to the in-flight crew about possible mechanical failures in the aircraft, the experience and qualifications of the pilot, expected weather conditions, etc. (2). Further, drinking alcohol or abusing other substances may be an attempt at coping with psychological distress but may also add to the potential for aggression or other antisocial behaviors (2, 4, 8, 17).

The implications for a sufferer of aviophobia may be profound and result in avoidance behavior, which is likely to create friction within personal relationships (2, 4, 18). Additionally, a high level of emotional distress and a sense of shame may be associated with flying (2, 4, 19). In addition to personal difficulties, career effects may be substantive and highly detrimental in these modern times of “global society”, as many professionals frequently need to travel for work, mainly within large countries and sometimes across the globe, to visit remote locations, overseas suppliers, business partners, and the like (4, 19, 20). It is apparent that experiencing severe distress from or avoiding air travel may be severely detrimental to both current operational efficiencies and longer-term careers. Although progressively diminishing budgets for air travel and continual improvements in online communications technology may have diminished the potential effects to some degree, it remains highly important to make in-person contacts in order to be personally present in far-flung locations for the purpose of optimizing important personal relationships and managing intercultural difficulties and similar issues (2, 4, 6).

Recent analysis using a perhaps more sophisticated, weighted approach to account for varying degrees of the disorder estimates an economic impact range between $19.64 million and $29.45 million a year (21). Further, disruptions due to time-consuming last-minute deplaning or even flights returning once airborne in extreme cases are highly costly. It should be noted, incidentally, that meeting the diagnostic criteria for aviophobia is not necessary in order for sufferers to experience detrimental effects or potentially benefit from psychological treatments for the disorder (2, 4, 6).

## Methods of aviophobia assessment

Several recent studies examining the efficacy of aviophobia assessment use several methods. Two crucial and widely utilized diagnostic tools previously mentioned are the Flight Anxiety Situations Questionnaire (FAS) and the Flight Anxiety Modality Questionnaire (FAM). A study conducted in 1999 (9), noting especially the importance of evaluating a sufferer’s “feelings, attitudes, and cognitions about specific flying related events” in cognitive behavioral therapy, examines the validity, reliability, and development of the two scales. The study’s authors note that the FAS includes “situations relevant to a journey by plane and generally connected with airplanes that can provoke anxiety”. The FAM, conversely, is much more oriented to assess different distressing thoughts and symptoms of anxiety or anticipatory anxiety in flight situations (4, 9, 22–25). Both instruments were assessed, and it was found that correlations for both test-retest validity and internal consistency were statistically significant at the.001 level. Convergent validity may, conversely, be evaluated with the Fear Survey Schedule (FSS-III), while divergent validity, correspondingly, may be evaluated by comparison with the Visible Analogue Flight Anxiety Scale (VAFAS) (9, 26).

In addition to these two widely used measures, other screening tools may be useful, such as the Trauma Symptoms Checklist (TSC-40), which may help to understand basic mechanisms of this syndrome based on individual histories mainly linked to various traumatic experiences and stress (27). In addition, a specific role may be played by the so-called stress-related sensitization to various stimuli that may be exaggerated and experienced too sensitively as dangerous or life-threatening (4, 28, 29). Other findings also indicate that stress and stress-related sensitization may influence limbic excitability or irritability, which may be linked to limbic or temporal lobe epileptiform activity, also called epileptic-like activity (28, 30–32).

These symptoms related to limbic and temporal epileptic-like or epileptiform activity were first described by Jackson in patients with temporal lobe epilepsy, and Jackson called them “dreamy states” as a response to an unknown neurological dysfunction (33, 34). Later these symptoms were also described as “temporal lobe lability” (35). In further research, Teicher called these epileptic-like symptoms “limbic irritability”, which can be measured by the LSCL-33 (Limbic System Checklist) questionnaire, and these epileptic-like activities, in the majority of cases, cannot be measured using usual EEG measures (36). These symptoms may usually present as cognitive, behavioral, affective, sensory, and memory disturbances, and they also may manifest as somatic or painful symptoms, mainly in patients with anxiety disorders (including aviophobia), affective disorders, posttraumatic stress disorder, and other mental disorders (28, 30, 36).

## Psychotherapeutic principles of aviophobia treatment

In 2003, Wiederhold and Wiederhold (37) conducted a longitudinal study of the recipients of varying treatment options. Coincidentally, the follow-up occurred only a few months after the 9/11/2001 terrorist attack, which occurred in New York City, and this study reported successful treatment results in many cases, mainly using Eye Movement Desensitization and Reprocessing (EMDR) and cognitive behavioral therapy that demonstrated sustainable benefit.

Contemporary findings indicate that cognitive behavioral therapy, mainly focused on “desensitization”, provides effective treatment options for aviophobia (4, 38–40). The most effective of these cognitive behavioral methods based on usual principles of exposure seems to be using virtual reality simulations, which are becoming progressively more affordable and available to clinicians as a treatment method (38, 41, 42). For example, a study by Glanz et al. (43) indicates “treatment in an office, with virtual airplanes at virtual airfields, accompanied by the noise of engines and other aspects of the flying experience, constitutes a highly desirable alternative”.

Recently, aviophobia research has undergone significant shifts primarily focused on improving the efficacy, accessibility, and personalization of treatment modalities such as Virtual Reality Exposure Therapy (VRET). Now, virtual reality represents a good standard used for controlled therapeutic exposure to aviation-related stressors such as turbulence, takeoff and landing, and other stressful situations, and it is also usually associated with measurement of psychophysiological responses. In addition, these successfully adapted aviophobia treatment protocols also use low-cost mobile virtual reality (VR) headsets paired with structured, unguided or minimally guided smartphone apps. These studies consistently demonstrate a significant reduction in overall anxiety symptoms, and these approaches may be used as a low-threshold intervention before a patient requires traditional psychotherapy (44–47). Another central trend in treating phobias involves using game design to substitute for or augment the role of an in-person therapist, packaging the therapeutic cognitive-behavioral framework directly into an immersive game loop presenting the real-life experience as a serious game with a psychotherapeutic goal rather than intimidating clinical treatment, considerably improving user engagement and leading to a statistically significant reduction in symptoms measured by the Flight Anxiety Situations Questionnaire (FAS), with a large effect size and mimicking the systematic desensitization needed for phobia control (48, 49).

These “real life” simulations related to virtual reality, in addition, seem to be effective for a longer time after treatment. For example, Rothbaum et al. (41, 42 examined the efficacy of this therapy in participants, divided into “WL” (wait-list control group), “virtual reality” (VRE), and “standard exposure” (SE) groups. The study indicated, for both VRE and SE exposures, equal treatment efficacy in symptom reduction, as measured by both standardized scales and actual ability to utilize commercial air travel. Subsequent longitudinal measurement collected one year later identified that 80% of the subjects from both VRE and SE retained their improvements (92 and 91%, respectively) in symptom reduction relative to baseline. Potentially noteworthy, however, is that a substantial percentage of the VRE participants did report use of alcohol or drugs during their flights, which may imply self-medication (42).

Additional recent research findings related to virtual reality treatment indicate that treatment outcomes from VRE treatment for aviophobia administered remotely via the internet do not differ significantly from those of face-to-face treatment in a clinician’s office (50). Another study shows that the inclusion of diaphragmatic breathing, a relaxation technique in VR exposure therapy, does not interfere with treatment outcomes and may enhance them, although results indicating an increase in efficacy were not significant (51).

According to a meta-analysis of 38 studies, virtual reality simulation of flight experience (VRE) does sufficiently mimic *in vivo* experience to result in psychophysiological arousal, particularly as measured by electrodermal activity; however, evidence regarding habituation remains inconclusive (52). Another meta-analysis of 11 studies suggests that the efficacy of virtual reality exposure is comparable to, or possibly greater than, that of traditional exposure-based interventions (53). A subsequent meta-review of meta-analyses focused on the general efficacy of virtual reality-based treatment further showed that VRE as a whole is not superior to other evidence-based treatments; however, in aviophobia, responses to active control conditions appear more favorable and conditioning effects may remain more stable over time (44–47, 54).

Other recent findings indicate that lower levels of self-reported anxiety during *in vivo* flight exposure are the best predictor of successful long-term exposure therapy outcomes (55); that the degree of “synchrony” (the magnitude of agreement between subjective emotional experience and a physiological measure) is not related to the degree of the phobic stimulus and does not predict treatment outcome (39); that the use of general cognitive coping strategies (e.g., self-monitoring, progressive muscle relaxation) at baseline is not predictive of long-term outcomes in self-reported flight anxiety and in-flight behavior, although the use of adaptive (e.g., seeking social support) coping strategies predicts an increase of maladaptive coping and, even more so, avoidance of maladaptive (e.g., self-medication) coping (56); that a moderating relationship appears to exist among anxiety sensitivity, experienced somatic sensations, and in-flight anxiety (57); and that ‘flying-phobic’ individuals (those scoring high on flight anxiety scales) estimate the probability of both general and negative flight events significantly higher than do ‘non-flying phobics’ (those scoring very low on flight anxiety scales), and have temporal orientation scores, as measured by the Zimbardo Time Perspective Inventory, that are higher in ‘Past-negative’ and lower in ‘Present-Hedonistic’, both aversive to the Inventory’s identified ideals for these measures (58).

There are, of course, other treatment options beyond cognitive behavioral therapy. One study (59) evaluated the efficacy of Eye Movement Desensitization and Reprocessing (EMDR) administered in parallel with cognitive behavioral therapy. The EMDR method developed by Francine Shapiro in the 1980s is one of the most frequently used treatment methods for posttraumatic stress disorder (59–64). Psychological distress is created by disturbing and traumatic aversive experiences, and EMDR decreases emotional tension by utilizing ‘stimulating’ lights, noises, or motions to create a distraction for the mind while the sufferer visualizes the negative experience, which induces the sufferer’s brain to “reprocess the thought without experiencing the negativity that was once associated with it” (65). It should be noted within this context that EMDR’s empirical validation for use in phobias is limited, and limited results have been reported for the treatment of panic disorder and agoraphobia (59, 66). In Triscari’s research, subjects were randomly assigned to groups for treatment with ‘behavioral conditioning’ or EMDR, respectively. For ten weeks, both groups were provided with psycho-educational treatments in two-hour sessions, including information about aviation safety and anxiety, instruction in cognitive behavioral relaxation techniques, and the re-characterization of dysfunctional thoughts. Subsequently, the subjects were divided into their groups, and the EMDR treatment group was administered EMDR treatment in individual psychotherapy sessions. The session focused on the subject’s earliest recalled perception that death or bodily harm may result from commercial flight and associated emotions. Following this, all subjects were gathered to receive additional cognitively oriented exposures, including a “real life” flight and a visit to an air traffic control tower. Subsequently the FAM and FAS were administered to the subjects. No significant difference was able to be identified between the behavioral conditioning group and the EMDR group. This suggests that EMDR is a viable treatment option for aviophobia. It is further noteworthy that more than 90% of the participants were able to fly on a commercial flight following treatment (59). Consistent with a similar result found by a preceding study (41, 42), it appears that 90% or more is an achievable and expected standard for treatments for aviophobia. Triscari et al. (67) later found equivalent treatment efficacy in a comparison between cognitive behavioral therapy individually integrated with systematic desensitization, EMDR, and VRE.

Another alternative for future research on aviophobia treatment may be meditation methods. These meditative practices, such as the well-known mindfulness method, may be performed with the intention of acknowledging a cognitive content without judgments and may include “emptying the mind” and relaxation, which may be helpful for coping with aviophobia-related emotional states (68–73). Using mindfulness to treat aviophobia bridges the gap between cognitive-behavioral techniques and central nervous system regulation and enables individuals to learn how to tolerate their “own internal feelings” and related somatic responses using metacognitive awareness, which represents the ability to observe transient mental events and view them as episodic moments rather than “absolute facts”. In addition, contemporary treatment approaches, including mindfulness-based cognitive-behavioral therapy (74, 75) and mindfulness-based stress reduction, may provide very useful treatment alternatives with significant effects on the reduction of anxiety, depression, and stress and on the general improvement in quality of life (68, 75–81).

## Conclusion and perspectives for further research and treatment

As previously mentioned, stress-related epileptiform activity may also manifest in some cases of aviophobia (31). In addition, there is evidence that the effects of stress may result in various neurophysiological and morphological changes (28, 31, 82); for example, stress may cause decreased volume of the hippocampus and corpus callosum, other alterations in the frontal and temporal lobes, and reduced neuronal density in the anterior cingulate cortex (28, 83–92).

It seems significant that mindfulness in the treatment of aviophobia (and other stress-related mental disorders) may have positive effects on various neurobiological processes and may improve pathological stress-related alterations and brain morphological changes (29, 93, 94). Furthermore, epileptiform activity may represent a specific physiological mechanism mediating the relationship between stress and other neurophysiological processes which may be influenced by practicing mindfulness. For example, research indicates that applying mindfulness may influence cortical thickness and gray matter concentration and volume and thus may also be a useful treatment method in various phobia cases (93, 95–99).

Within this context, mindfulness and mindfulness-based therapies might represent – alongside or integrated into the already proven and established treatment methods mentioned in this article, such as cognitive behavioral therapy, VRE, or EMDR – a promising opportunity for future research and a treatment option for aviophobia.
